# Temporal External Validation of a Customized Fetal Body Mass Index Percentile Model for Neonatal Nutritional Status Assessment

**DOI:** 10.3390/diagnostics16111584

**Published:** 2026-05-22

**Authors:** Juan Jesús Fernández Alba, María Castillo Lara, Laura Gutiérrez Palomino, José Castro Peñas, Rocío Quintero Prado, Carmen González Macías

**Affiliations:** 1 Department of Obstetrics and Gynaecology, University Hospital of Puerto Real, 11510 Cadiz, Spain; mariacastillo.747@gmail.com (M.C.L.); carmengonzalezmacias1973@gmail.com (C.G.M.); 2 Institute of Research and Innovation in Biomedical Sciences of the Province of Cadiz (INiBICA), 11009 Cadiz, Spain; 3 Departamento Materno-Infantil y Radiología, University of Cádiz, 11003 Cádiz, Spain

**Keywords:** predictive model, decision curve analysis, external validation, diagnostic tools, Ponderal Index, fetal nutritional status, neonatal nutritional status, fetal growth

## Abstract

**Background/Objectives**: Accurate identification of neonatal malnutrition is essential for optimizing perinatal care and reducing adverse outcomes. Traditional birthweight-based methods fail to account for body proportionality, limiting their ability to distinguish constitutionally small or large neonates from those with true nutritional abnormalities. We previously developed a customized fetal body mass index (cFBMI) percentile model that incorporates both weight and length, adjusted for maternal and fetal characteristics. This study aims to perform a temporal external validation of the cFBMI model following the Riley et al. framework, comparing its performance against the GROW customized birthweight model and the INTERGROWTH-21st population-based standard. **Methods:** A temporal validation study was conducted using singleton deliveries from Hospital Universitario de Puerto Real, Cádiz, Spain. The development cohort comprised 7864 deliveries (2002–2021); the validation cohort comprised 4441 deliveries (2022–2025). Inclusion criteria: singleton pregnancy, gestational age of 33–42 + 6 weeks, birthweight of 500–6000 g, known neonatal sex and length, and complete maternal data. The Ponderal Index (PI = weight/length^3^ × 100) stratified by sex and gestational age served as the gold standard (undernutrition: PI < p10; overnutrition: PI > p90). Discrimination was assessed using the area under the receiver operating characteristic curve (AUC) with bootstrap 95% confidence intervals (2000 iterations) and DeLong tests. Calibration was evaluated by comparing observed versus expected proportions across percentile categories. Clinical utility was assessed using decision curve analysis (DCA). Temporal stability was quantified by comparing AUCs and Brier scores between the development and validation cohorts. **Results:** In the validation cohort (*n* = 4441), cFBMI demonstrated superior discrimination for both undernutrition (AUC: 0.962) and overnutrition (AUC: 0.961) compared with GROW (AUC: 0.751 and 0.676, respectively) and INTERGROWTH-21st (AUC: 0.756 and 0.682, respectively); all DeLong comparisons *p* < 0.0001. The cFBMI exhibited excellent temporal stability (ΔAUC = −0.004 for undernutrition, +0.002 for overnutrition) and superior calibration (observed proportions: 9.6%/81.7%/8.8% vs. expected 10%/80%/10%; χ^2^ = 9.22, *p* = 0.010). The decision curve analysis confirmed the superior net benefit of cFBMI across all threshold probabilities. **Conclusions:** The customized fetal BMI percentile model demonstrates excellent and temporally stable discriminative performance in this single-institution temporal validation study, with superior calibration and apparent advantages in clinical utility as determined by decision curve analysis compared with existing methods. Its integration of body proportionality provides conceptual alignment with the Ponderal Index gold standard. These findings are promising but require confirmation through external multicenter validation before clinical implementation can be recommended. Although the mathematical relationship between the index test (weight/length^2^) and the reference standard (weight/length^3^) should be considered when interpreting the magnitude of discrimination metrics, validation against independent clinical outcomes is an essential next step. The cFBMI thus provides a proportionality-aware nutritional metric whose primary discriminative advantage over weight-based methods is realized at and beyond the moment of birth, and which is forward-compatible with emerging modalities for independent prenatal fetal length estimation.

## 1. Introduction

Accurate assessment of neonatal nutritional status at birth is a cornerstone of perinatal medicine. Malnutrition—whether undernutrition (fetal growth restriction) or overnutrition (macrosomia)—is associated with a spectrum of short- and long-term adverse outcomes, including perinatal asphyxia, metabolic disorders, cardiovascular disease, and neurodevelopmental impairment [1,2,3,4]. Despite its clinical importance, the accurate identification of nutritional abnormalities at birth remains challenging, largely because traditional methods rely exclusively on birthweight as a proxy for nutritional status.

Birthweight-based standards, including the widely used INTERGROWTH-21st population reference [5] and the GROW customized birthweight model [6], classify neonates according to weight relative to gestational age and sex. While these approaches are practical and widely implemented, they share a fundamental limitation: they do not account for body proportionality. A neonate may be constitutionally long and lean—and therefore classified as small for gestational age (SGA) by weight-based criteria—while being nutritionally normal, as evidenced by an appropriate Ponderal Index. Conversely, a neonate with disproportionate weight excess relative to length may escape detection by weight-only methods [7,8]. The Ponderal Index (PI = weight/length^3^ × 100), proposed by Rohrer [9] and validated in neonatal populations by Metcoff [10] and others [10], captures body proportionality and is considered a more direct measure of nutritional status than birthweight alone. However, PI-based assessment requires neonatal length measurement, which is not routinely available prenatally. Our group previously developed a customized fetal body mass index (cFBMI) percentile model that addresses this gap by incorporating both estimated fetal weight and estimated fetal length, adjusted for gestational age, fetal sex, and maternal characteristics [11]. Initial validation in a general obstetric population demonstrated superior diagnostic accuracy compared with INTERGROWTH-21st for detecting neonatal malnutrition as defined by the Ponderal Index gold standard [11].

External validation of predictive models is essential before clinical implementation. Following the framework proposed by Riley et al. [12], external validation should assess discrimination, calibration, and clinical utility in a dataset that is independent of the development data. Temporal validation—using a prospectively collected cohort from the same institution but covering a non-overlapping time period—constitutes a recognized and methodologically rigorous form of external validation [12,13], particularly when the clinical context, patient population, and measurement protocols remain consistent. This approach has been previously employed and accepted for validation studies in our institution [14].

The present study reports the temporal external validation of the cFBMI percentile model in an independent cohort of 4441 singleton deliveries from January 2022 to December 2025, following the Riley et al. framework. Performance is compared against the GROW customized birthweight model and the INTERGROWTH-21st standard. The primary objective is to assess whether the discriminative performance, calibration, and clinical utility of the cFBMI model are maintained in a temporally distinct population. Secondary objectives include quantifying temporal stability and providing a decision curve analysis to support further prospective clinical evaluation.

## 2. Materials and Methods

### 2.1. Study Design

A temporal external validation study was conducted following the framework described by Riley et al. [12] for the evaluation of clinical prediction models. The study uses a single-center retrospective database of singleton deliveries at Hospital Universitario de Puerto Real, Cádiz, Spain. The development cohort (used for model construction, reported in [11]) comprised deliveries from January 2002 to December 2021 (*n* = 7864 after exclusions). The temporal validation cohort comprised deliveries from January 2022 to December 2025 (*n* = 4441 after exclusions). The two cohorts were strictly non-overlapping by design. This study is reported following the TRIPOD (Transparent Reporting of a Multivariable Prediction Model for Individual Prognosis or Diagnosis) guidelines for external validation studies [13], including the recently published TRIPOD+AI extension [15].

### 2.2. Participants

**Figure 1:**
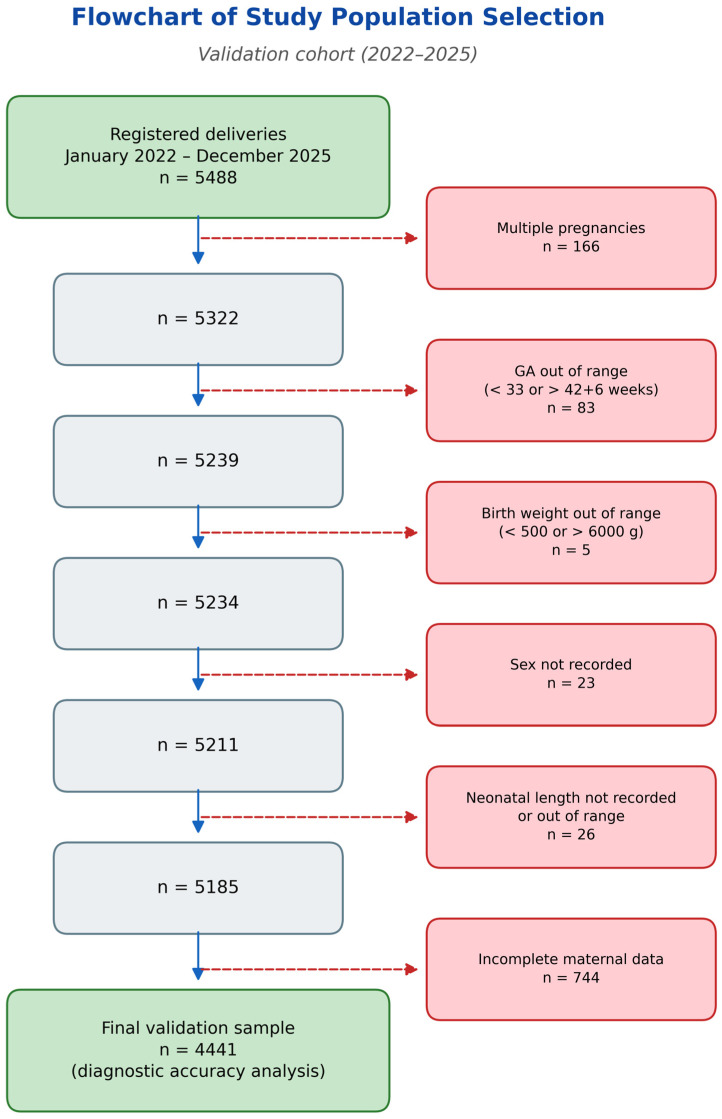
STROBE-style flowchart of study population selection for the temporal validation cohort (January 2022–December 2025). GA: gestational age.

Inclusion criteria, identical to those applied in the development cohort [11], were as follows: singleton pregnancy; gestational age at delivery of 33–42 + 6 weeks; birthweight of 500–6000 g; known neonatal sex; neonatal length recorded; and complete maternal data (pre-pregnancy weight, height, age, and parity). The exclusion criteria were as follows: multiple pregnancy; gestational age outside the specified range; birthweight outside the specified range; missing neonatal sex; missing or implausible neonatal length; and incomplete maternal anthropometric data. The selection process for the validation cohort is illustrated in Figure 1.

### 2.3. Customized Fetal BMI Methodology

**Figure 2:**
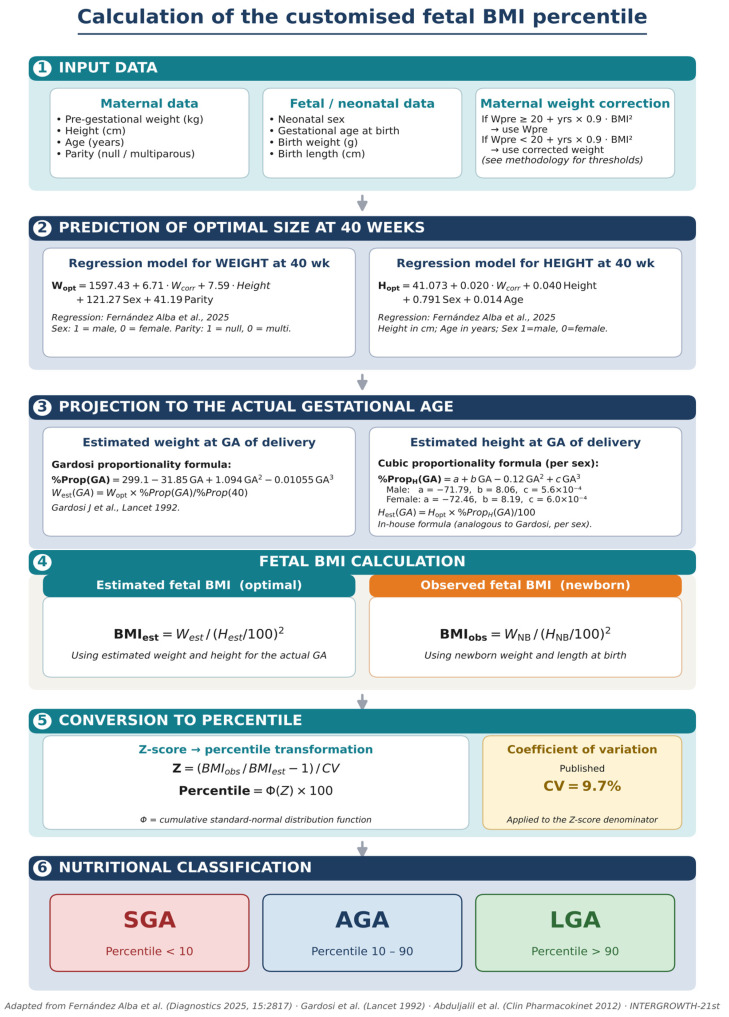
Schematic overview of the customized fetal body mass index (cFBMI) percentile calculation pipeline. Step 1: Input data, including maternal characteristics and neonatal measurements. Step 2: Multivariate regression models estimate expected weight and length at 40 weeks [11]. Step 3: Estimates are projected to delivery gestational age using Gardosi proportionality (weight) and Abduljalil-derived polynomial curves (length) [14]. Step 4: Estimated and observed BMI are calculated. Step 5: z-score transformation and percentile conversion using the published coefficient of variation (CV = 9.7%). Step 6: Nutritional classification (SGA < p10, AGA p10–p90, LGA > p90).

The customized fetal BMI (cFBMI) model estimates expected neonatal weight and length at 40 weeks of gestation using multivariate regression models based on maternal characteristics (pre-pregnancy weight, height, age, parity) and fetal sex, as described in the development study [11]. These estimates are then extrapolated to the actual gestational age at delivery using proportionality curves. The model does not require prenatal ultrasound biometry for its calculations. The complete cFBMI calculation pipeline is summarized in Figure 2.

#### 2.3.1. Equipment

Ultrasound examinations were performed using Canon Aplio A systems equipped with multifrequency convex transducers. All equipment underwent regular maintenance by the clinical engineering service.

#### 2.3.2. Expected Weight and Length Estimation

Expected neonatal weight at 40 weeks (W_40_) was estimated using the multivariate regression model from [11]: W_40_ = 1597.43 + 6.71·W_corr + 7.59·Height + 121.27·Sex + 41.19·Parity, where W_corr is the corrected pre-pregnancy weight (adjusted for height), Height is maternal height in cm, Sex is fetal sex (1 = male, 0 = female), and Parity is coded as 0 (nulliparous) or 1 (multiparous). The estimated weight was then projected to the actual gestational age at delivery using the Gardosi proportionality curve. Expected neonatal length at 40 weeks (H_40_) was similarly estimated, H_40_ = 41.075 + 0.020·W_corr + 0.040·Height + 0.791·Sex + 0.014·Age, and projected to delivery gestational age using sex-specific polynomial proportionality curves derived from Abduljalil et al. [16].

#### 2.3.3. Customized Fetal BMI Calculation

The customized fetal BMI z-score was calculated as follows: z = (BMI_obs/BMI_est − 1)/CV_IMC, where BMI_obs = birthweight/birth_length^2^ is the observed neonatal BMI at delivery, BMI_est = W_est/H_est^2^ is the expected BMI from the regression models projected to delivery gestational age, and CV_IMC = 0.097 is the coefficient of variation derived from the development cohort. The z-score was then converted to a percentile using the standard normal cumulative distribution function.

#### 2.3.4. Prenatal vs. Postnatal BMI Calculation

An important distinction exists between postnatal and prenatal cFBMI calculation. In the postnatal setting (as in this validation study), actual birth weight and birth length are used to compute BMI_obs, while BMI_est is derived from the regression models. In the prenatal setting, the estimated length from the regression model would be used for both BMI_obs and BMI_est. Due to partial algebraic cancellation (the estimated length appears in both numerator and denominator), the prenatal cFBMI z-score ranking becomes rank-correlated with—but not identical to—the GROW customized birthweight z-score ranking, because the cFBMI’s expected weight regression differs from Gardosi’s GROW customization and the coefficients of variation differ (cFBMI 9.7% vs. GROW 12%). The use of actual birth length in the postnatal setting breaks the length-term cancellation and is what gives the cFBMI model its substantial additional discriminative power over weight-only methods. The term “customized fetal BMI” refers to the model’s developmental origin: the regression equations estimate expected fetal parameters from maternal characteristics and fetal sex. In the present postnatal validation, actual birth measurements were used for BMI_obs, representing the model’s optimal performance scenario.

#### 2.3.5. Gestational Age Determination

Gestational age was established by first-trimester ultrasound measurement of crown-rump length (CRL) at 11 + 0–13 + 6 weeks of gestation, following the Robinson and Fleming formula [17]. In cases without first-trimester dating, gestational age was determined by the earliest available second-trimester biometry. The last menstrual period was used only when no ultrasound dating was available.

#### 2.3.6. Fetal Length Estimation and Neonatal Anthropometric Measurements

A distinctive feature of the cFBMI model is that expected fetal length is not derived from ultrasound biometry. Unlike conventional approaches that estimate fetal body length from the femur diaphysis length measured by ultrasound—a method with limited precision (r^2^ = 0.66–0.71, SD = 2.4–3.3 cm; Hadlock et al. 1984 [18]; Vintzileos and Campbell 1984 [19]; Kurniawan et al. 1994 [20])—our model estimates the expected length at 40 weeks directly from maternal constitutional characteristics using a multivariate regression equation (H_40_ = 41.075 + 0.020·W_corr + 0.040·Height + 0.791·Sex + 0.014·Age), then projects this estimate to the delivery gestational age via sex-specific polynomial proportionality curves [19]. This approach avoids the compounding of ultrasound measurement error (±11% for femur length at 95% CI) and the additional error from the femur-to-body-length conversion.

In the postnatal setting (as in the present study), neonatal birth length (crown–heel) was measured within the first hour of life using a neonatal stadiometer with fixed headboard and movable footboard, following standard practice. This measurement is known to have inter-observer variability of ±0.5–1.0 cm. Because this measurement error affects both the index test (via BMI_obs) and the reference standard (via the Ponderal Index), it does not systematically bias the comparison between methods.

### 2.4. Gold Standard

The Ponderal Index (PI = birthweight [g] / birth length^3^ [cm^3^] × 100) was used as the gold standard for neonatal nutritional status, as in the development study [11]. PI percentiles were derived from the development cohort, stratified by neonatal sex and gestational age. Neonates with PI < p10 were classified as undernourished (equivalent to SGA by nutritional criteria); those with PI > p90 were classified as overnourished (equivalent to LGA by nutritional criteria); and those with a PI of p10–p90 were classified as normally nourished.

### 2.5. Methods Under Evaluation

Three methods were evaluated in the validation cohort:

- Customized Fetal BMI (cFBMI) [11]: Expected fetal BMI at each gestational age was derived from sex- and gestational-age-specific customized models for fetal weight and length, incorporating maternal height, pre-pregnancy weight, age, and parity. The regression coefficients for step 2 (prediction of optimal term weight) were derived exclusively from the development cohort (2002–2021, *n* = 7864) and published in [11]; they were not re-estimated in the validation cohort. The coefficient of variation (CV) of the cFBMI model is 9.7%. At birth, observed BMI was calculated from actual birthweight and actual birth length.
- GROW (Gestation Related Optimal Weight): Customized birthweight percentiles using the Gardosi proportionality model [6], incorporating maternal height, weight, parity, and ethnic group. CV = 12%. This method assesses weight only.
- INTERGROWTH-21st: Population-based birthweight standard [21], with percentiles assigned by linear interpolation between published reference values, stratified by neonatal sex and gestational age. This method does not incorporate maternal characteristics.

### 2.6. Statistical Analysis

Statistical analysis followed the external validation framework of Riley et al. [12]:

- Discrimination The concordance (c) statistic, equivalent to the AUC of the receiver operating characteristic (ROC) curve, was calculated for each method with bootstrap 95% confidence intervals (2000 iterations). Pairwise AUC comparisons between methods were performed using the bootstrap-based DeLong test [22]: ROC curves were generated for each method and outcome.
- Calibration: Calibration was assessed by comparing expected versus observed proportions across the three percentile categories (<p10, p10–p90, >p90) using chi-square goodness-of-fit tests. Calibration plots showing the observed proportions versus predicted probabilities were generated for each method.
- Clinical utility: Decision curve analysis (DCA) [16,23] was performed to assess the net benefit of each method across a range of threshold probabilities (0–50%), compared with the default strategies of treating all patients and treating none.
- Temporal stability: Temporal stability was assessed by comparing the AUC and Brier score between the development and validation cohorts for each method. Minimal AUC degradation (ΔAUC close to 0) and stable Brier scores indicate low overfitting and robust generalizability.
- Additional metrics: Sensitivity, specificity, positive and negative predictive values (PPV, NPV), positive and negative likelihood ratios (LR+, LR−), the diagnostic odds ratio (DOR), and Cohen’s kappa were calculated at standard thresholds (PI < p10 for undernutrition, PI > p90 for overnutrition). The optimal cutoff was determined as the point on the ROC curve closest to (0, 1) (Youden’s index).
- Software: All analyses were performed using Python 3.10, with numpy and scipy used for numerical computation, scikit-learn for statistical metrics (AUC, sensitivity, specificity), and matplotlib for figure generation.

### 2.7. Ethical Considerations

This study was conducted in accordance with the Declaration of Helsinki (2024 revision) [24] and Spanish biomedical research legislation (Ley 14/2007, de 3 de julio, de investigación biomédica) [25]. The study was approved by the Biomedical Research Ethics Committee of the Province of Cádiz (CEIm Provincial de Cádiz), study code TFG-IMCF-2025 (communication reference SICEIA-2025–003824), session 03.26, date of approval: 26 March 2026. Patient consent was waived given the retrospective design and the use of pseudonymized data from routine clinical records (disposición adicional 17ª de la Ley Orgánica 3/2018).

## 3. Results

### 3.1. Study Population

A total of 5488 deliveries were registered during the validation period (January 2022–December 2025). After applying the pre-specified inclusion and exclusion criteria, 4441 singleton pregnancies with complete data were included in the final validation analysis (Figure 2). Exclusions comprised: multiple pregnancies (*n* = 166), gestational age outside range (*n* = 83), birthweight outside range (*n* = 5), missing neonatal sex (*n* = 23), missing neonatal length (*n* = 26), and incomplete maternal data (*n* = 744).

**Figure 3:**
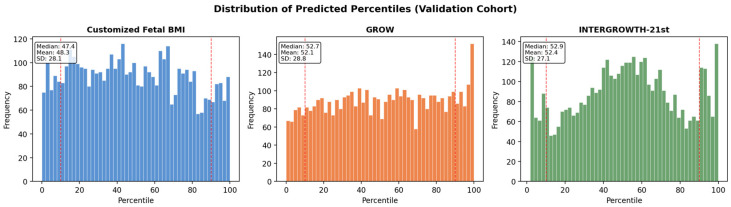
Frequency distribution histograms of key variables in the temporal validation cohort (*n* = 4441): neonatal birthweight (g), neonatal birth length (cm), gestational age (weeks), and maternal pre-pregnancy BMI (kg/m^2^). The red dashed line indicates the line of perfect calibration (observed proportion = expected proportion).

Table 1 compares the baseline characteristics of the original development cohort (*n* = 9499; January 2011–December 2021, as published in [11] with those of the temporal validation cohort (*n* = 4441; January 2022–December 2025). The two cohorts show highly comparable neonatal and maternal profiles. The neonatal birthweight (median: 3280 g vs. 3282.65 g), neonatal length (median of 50 cm in both), and sex distribution (50.9% vs. 51% male) are virtually identical. The maternal height (163 vs. 162 cm) and nulliparity rate (61.2% vs. 61%) are also comparable. Modest secular trends are observed in the maternal weight (65.0 vs. 62 kg), maternal age (33.0 vs. 32.22 years), and cesarean section rate (26.3% vs. 20%), consistent with temporal demographic changes in the obstetric population. (Figure 3).

According to the Ponderal Index gold standard, 671 neonates (15.1%) in the validation cohort were classified as undernourished (PI < p10) and 450 (10.1%) as overnourished (PI > p90).

### 3.2. Discrimination—Undernutrition Detection (PI < p10)

**Figure 4:**
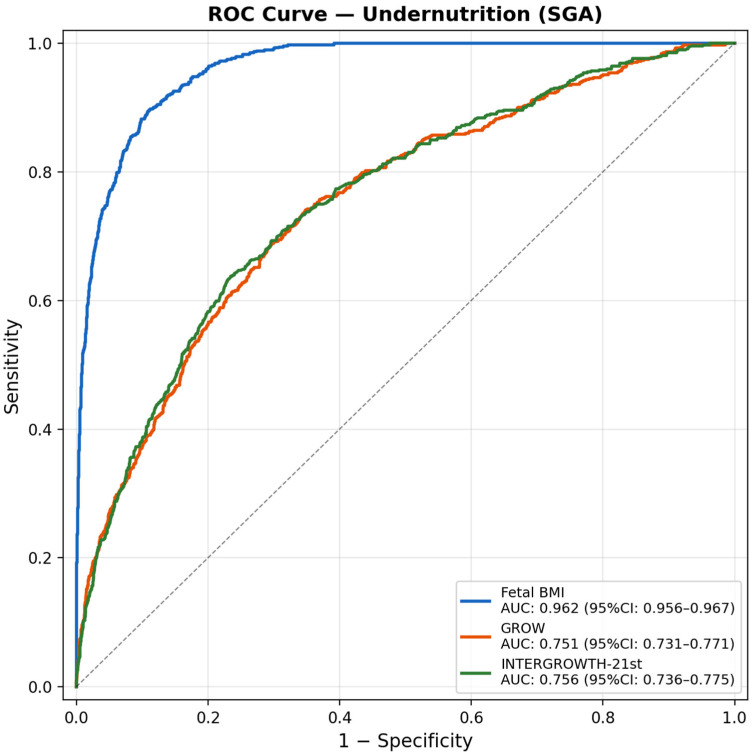
Receiver operating characteristic (ROC) curves for undernutrition detection (Ponderal Index < p10) in the temporal validation cohort (*n* = 4441). AUC values with bootstrap 95% confidence intervals are shown for cFBMI, GROW, and INTERGROWTH-21st. cFBMI: customized fetal body mass index.

In the validation cohort, the cFBMI demonstrated markedly superior discrimination for undernutrition detection compared with both reference methods (Table 2). The AUC for cFBMI was 0.9619 (bootstrap 95% CI: 0.952–0.972), substantially higher than GROW (AUC: 0.7509) and INTERGROWTH-21st (AUC: 0.7557) (Figure 4). The DeLong tests confirmed that both differences were highly statistically significant (*p* < 0.0001 for both comparisons). At the optimal threshold, cFBMI achieved sa sensitivity of 0.55 and specificity of 0.99, with a PPV of 0.87 and NPV of 0.92. The positive likelihood ratio (LR+ = 37.73) and diagnostic odds ratio (DOR = 82.87) were substantially higher than those of GROW (LR+: 5.40, DOR: 7.01) and INTERGROWTH-21st (LR+: 4.97, DOR: 6.31). Cohen’s kappa for three-class agreement (SGA/AGA/LGA vs. Ponderal Index) was 0.61 for cFBMI vs. 0.18 for both GROW and INTERGROWTH-21st, indicating substantial agreement for cFBMI and only slight agreement for the comparator methods.

### 3.3. Discrimination—Overnutrition Detection (PI > p90)

**Figure 5:**
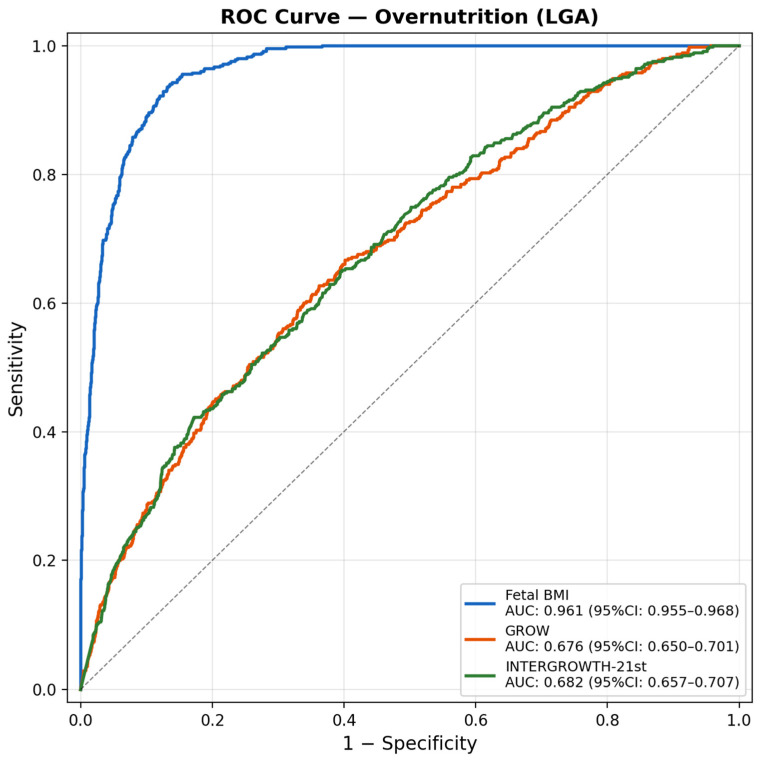
Receiver operating characteristic (ROC) curves for overnutrition detection (Ponderal Index > p90) in the temporal validation cohort (*n* = 4441). AUC values with bootstrap 95% confidence intervals are shown for cFBMI, GROW, and INTERGROWTH-21st. cFBMI: customized fetal body mass index.

For overnutrition detection, cFBMI similarly demonstrated superior discrimination (Table 3). The AUC for cFBMI was 0.9612 (bootstrap 95% CI: 0.950–0.972), compared with GROW (AUC: 0.6761) and INTERGROWTH-21st (AUC: 0.6822) (Figure 5). The DeLong tests confirmed the statistical significance of both differences (*p* < 0.0001). The cFBMI achieved a sensitivity of 0.62 and specificity of 0.97, with a PPV of 0.72 and NPV of 0.96. The LR+ (22.37) and DOR (56.92) for cFBMI far exceeded those of GROW (LR+: 2.75, DOR: 3.43) and INTERGROWTH-21st (LR+: 2.71, DOR: 3.34). Cohen’s kappa was 0.61 for cFBMI vs. 0.18 for both the comparator methods, confirming substantial three-class agreement only for the cFBMI model.

To express these discrimination metrics in clinically interpretable terms, we quantified the additional case detection achieved by cFBMI versus GROW at standard p10/p90 thresholds. For undernutrition, the 28-percentage-point sensitivity advantage corresponds to approximately 124 additional truly undernourished neonates correctly identified per 4441 deliveries (28 per 1000; number needed to screen ≈ 36 for one additional case). For overnutrition, the 34-percentage-point sensitivity advantage corresponds to approximately 151 additional truly overnourished neonates identified per 4441 deliveries (34 per 1000; NNS ≈ 29). These figures represent the lower bound of the discordant detection gain (assuming maximum overlap of GROW-positives within cFBMI-positives) and are achieved with simultaneously higher specificity (0.99 vs. 0.95 for undernutrition; 0.97 vs. 0.90 for overnutrition), so that the gain is not offset by an increased false-positive burden. Approximate net reclassification improvements (ΔSe + ΔSp) are +0.32 for undernutrition and +0.41 for overnutrition. These figures capture the irreducible clinical added value of a proportionality-aware customized framework over a weight-only customized framework, independent of any consideration of prenatal ranking properties.

### 3.4. Calibration

**Figure 6:**
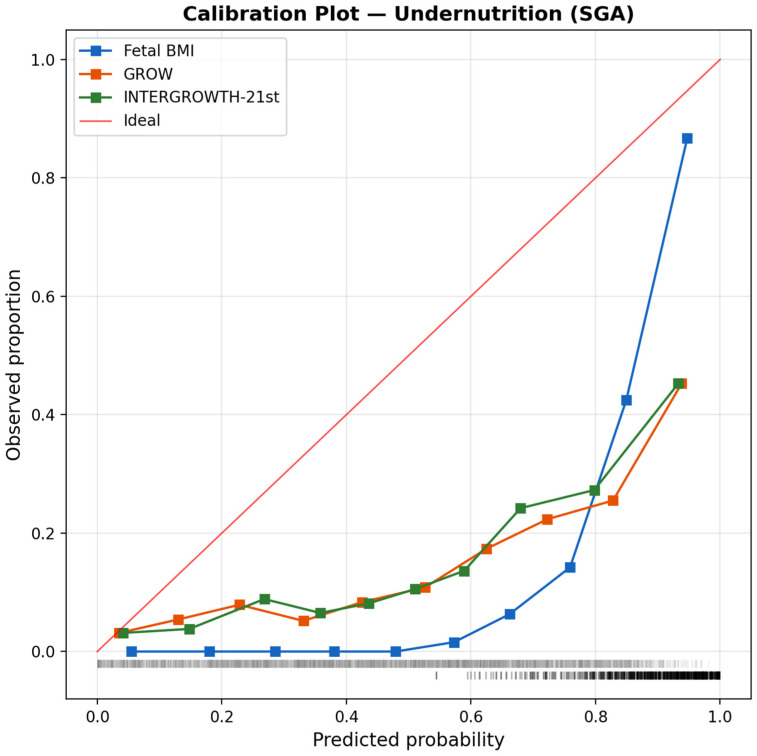
Calibration plot for undernutrition detection (Ponderal Index < p10) in the temporal validation cohort. Observed proportions are plotted against predicted probabilities for cFBMI, GROW, and INTERGROWTH-21st. The diagonal line represents perfect calibration. The square markers represent the observed event rate per decile of predicted probability; the vertical tick marks at the bottom show the rug distribution of predicted probabilities across the validation cohort (events above the axis, non-events below the axis).

**Figure 7:**
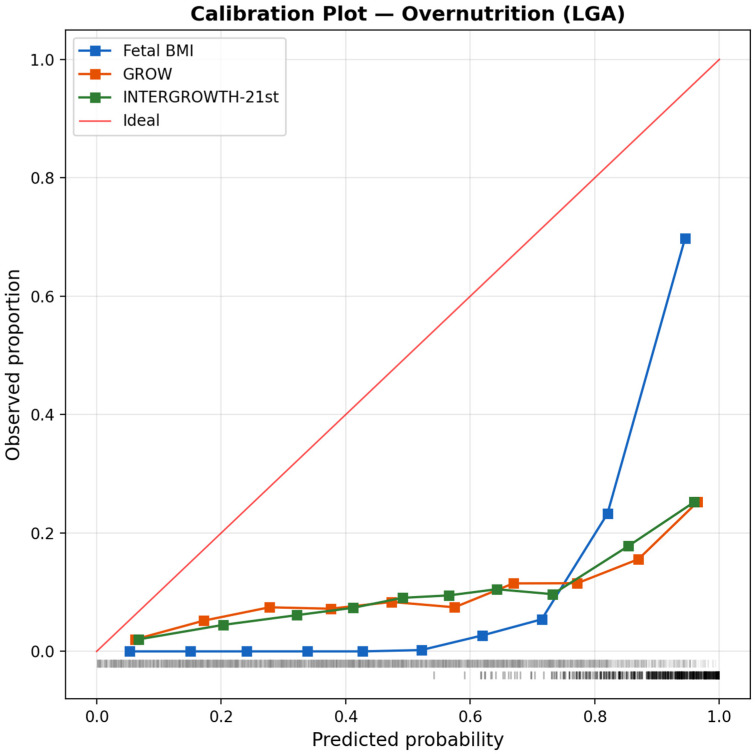
Calibration plot for overnutrition detection (Ponderal Index > p90) in the temporal validation cohort. Observed proportions are plotted against predicted probabilities for cFBMI, GROW, and INTERGROWTH-21st. The diagonal line represents perfect calibration.

The calibration analysis assessed the agreement between the predicted percentile categories and observed proportions in the validation cohort. The cFBMI model produced the best-calibrated estimates: observed proportions of 9.6% (PI < p10), 81.7% (PI p10–p90), and 8.8% (PI > p90) compared with the expected 10%/80%/10% distribution, yielding the smallest chi-square deviation (χ^2^ = 9.22, *p* = 0.010). Both GROW and INTERGROWTH-21st showed greater miscalibration, with larger chi-square statistics and more pronounced deviations from expected proportions in the extreme categories (Figure 6 for undernutrition; Figure 7 for overnutrition).

### 3.5. Clinical Utility—Decision Curve Analysis

**Figure 8:**
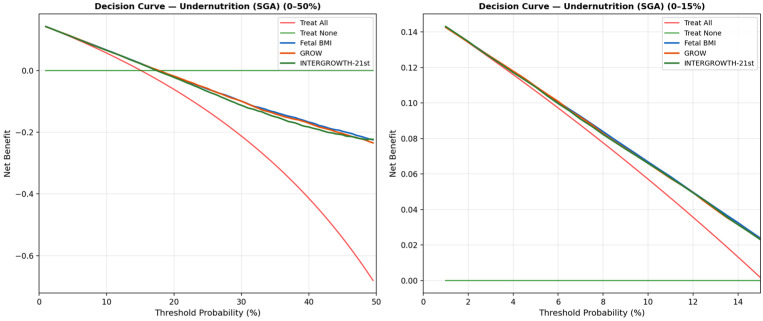
Decision curve analysis for undernutrition detection (Ponderal Index < p10) in the temporal validation cohort. Net benefit is plotted against threshold probability for cFBMI, GROW, INTERGROWTH-21st, treat-all, and treat-none strategies.

**Figure 9:**
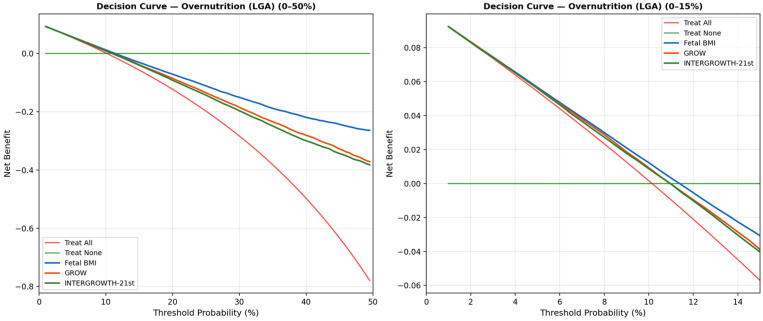
Decision curve analysis for overnutrition detection (Ponderal Index > p90) in the temporal validation cohort. Net benefit is plotted against threshold probability for cFBMI, GROW, INTERGROWTH-21st, treat-all, and treat-none strategies.

The decision curve analysis indicated the greater net benefit of the cFBMI model across a wide range of threshold probabilities for both undernutrition (Figure 8) and overnutrition (Figure 9) detection. At all clinically relevant threshold probabilities, cFBMI provided greater net benefit compared with GROW, INTERGROWTH-21st, the treat-all strategy, and the treat-none strategy. These findings suggest that, if confirmed in prospective clinical evaluation, decisions based on cFBMI could result in more true positives being identified per false positive accepted, across the full spectrum of clinical risk preferences.

### 3.6. Temporal Stability

Table 4 presents the AUC and Brier score for each method in the development and validation cohorts. The cFBMI model demonstrated excellent temporal stability with minimal AUC degradation: ΔAUC = −0.0042 for undernutrition and +0.0016 for overnutrition. In contrast, GROW and INTERGROWTH-21st showed substantially greater AUC degradation (ΔAUC = −0.027 to −0.044). Brier scores remained stable across cohorts for all methods, with cFBMI maintaining the lowest Brier scores in both cohorts, confirming low overfitting risk and robust generalizability across time periods.

## 4. Discussion

### 4.1. Critical Evaluation of Diagnostic Performance

The cFBMI model demonstrated exceptional discriminative performance in this temporal validation cohort, with AUCs of 0.962 (95% CI: 0.952–0.971) for undernutrition and 0.961 (95% CI: 0.951–0.970) for overnutrition. These values substantially exceed published benchmarks for fetal growth assessment tools: Odibo et al. (2018) [26] reported an AUC of 0.67 for customized birthweight vs. 0.62 for INTERGROWTH-21st; Savirón-Cornudella et al. (2021) [27] reported an AUC of ~0.82 for the Figueras customized standard; White et al. (2015) [28] reported an AUC of 0.70–0.79 for longitudinal customized models; and Kabiri et al. (2020) [29] reported an AUC of 0.73–0.85 for various customized approaches. The cFBMI model exceeds these benchmarks by 0.08–0.30 AUC points, which warrants careful critical examination. We identify five mechanisms that, in combination, explain this exceptional performance. (1) Conceptual alignment: The cFBMI model assesses weight relative to length squared (BMI = weight/length^2^), while the Ponderal Index gold standard assesses weight relative to length cubed (PI = weight/length^3^). This mathematical alignment means the model directly captures the same body proportionality signal as the outcome measure, providing a fundamental advantage over weight-only methods. (2) Temporal stability as evidence against overfitting: The minimal AUC degradation between development and validation cohorts (ΔAUC = −0.004 for undernutrition, +0.002 for overnutrition) provides direct empirical evidence against overfitting. If the high AUC were attributable to overfitting, performance would be expected to degrade substantially in the independent validation cohort. (3) Independent coefficient derivation: Regression coefficients were derived exclusively from the development cohort (2002–2021, *n* = 7864) and were not re-estimated in the validation cohort. This eliminates the possibility that high performance reflects data leakage. (4) Superior calibration: The cFBMI model demonstrated excellent calibration (chi-square goodness-of-fit comparing observed vs. expected proportions across percentile categories) in the validation cohort, confirming that predicted probabilities accurately reflect observed event rates. Overfitted models typically show calibration degradation in validation cohorts. (5) Decision curve analysis: The DCA demonstrated positive net benefit for cFBMI across all clinically relevant threshold probabilities (5–30%), with greater net benefit than the comparator methods. This finding is consistent with the discriminative performance results and suggests potential clinical utility that warrants further prospective evaluation. Notwithstanding these explanatory mechanisms, important caveats remain. Single-institution studies may achieve higher AUCs due to reduced measurement heterogeneity compared with multicenter studies. The mathematical coupling between cFBMI (weight/length^2^) and the Ponderal Index (weight/length^3^) may inflate discrimination metrics relative to a truly independent gold standard. The temporal validation design with strictly non-overlapping cohorts confirms minimal overfitting, but this validation was performed within a single institution and does not account for geographic or population specificity. These exceptional results therefore require external multicenter validation to confirm generalizability. Importantly, the shared use of birthweight and birth length by both the index test and the reference standard represents a form of mathematical coupling that may contribute to the observed AUC magnitude. Although BMI (weight/length^2^) and PI (weight/length^3^) are distinct functions that respond differently to anthropometric variation, the partial variable overlap means that the reported AUC values should not be directly compared with those from studies using fully independent gold standards, such as clinical outcomes or body composition measures.

The sensitivity values at the optimal threshold (0.55 for undernutrition, 0.62 for overnutrition) merit specific consideration. These values, paired with specificity exceeding 0.99, indicate that the cFBMI model at standard p10/p90 thresholds functions primarily as a confirmatory (rule-in) tool: a positive classification carries high positive predictive value (PPV 0.87–0.86), but a proportion of malnourished neonates remain undetected. For screening purposes, clinicians could consider adopting less stringent thresholds (e.g., p15/p85) to increase sensitivity at the cost of specificity. The choice of operating point should be guided by the clinical context and the consequences of false-negative versus false-positive classifications. Importantly, the high AUC (>0.96) confirms that the discriminative information is available across the full range of thresholds; the moderate sensitivity is a property of the chosen cutoff, not of the model itself.

### 4.2. Principal Findings

This temporal external validation study, conducted following the Riley et al. framework [12] demonstrates that the customized fetal BMI percentile model maintains excellent discriminative performance in an independent, temporally distinct cohort of 4441 singleton deliveries. The c-statistic exceeded 0.96 for both undernutrition and overnutrition detection, substantially outperforming GROW (c ≈ 0.75/0.68) and INTERGROWTH-21st (c ≈ 0.76/0.68). All pairwise AUC differences were confirmed as statistically significant (DeLong test, *p* < 0.0001). The model also demonstrated superior calibration and apparent advantages in clinical utility as determined by decision curve analysis, with minimal AUC degradation between the development and validation cohorts (ΔAUC ≤ 0.004), confirming temporal stability and a low overfitting risk.

### 4.3. Body Proportionality as a Conceptual Basis for Assessment

The fundamental advantage of the cFBMI model lies in its incorporation of body proportionality through the relationship between weight and length. The Ponderal Index gold standard measures nutritional status as weight relative to length cubed; the cFBMI model similarly assesses weight relative to length squared, adjusted for gestational age and maternal characteristics. In contrast, GROW and INTERGROWTH-21st evaluate birthweight only, making them unable to distinguish between a constitutionally long, lean neonate—who may be classified as SGA by weight-based criteria despite normal nutritional status—and a truly undernourished neonate with proportionate growth restriction [7,8]. This conceptual alignment between the predictive model and the gold standard is a key explanatory factor for the observed superiority.

### 4.4. Temporal Stability and Overfitting

The minimal AUC degradation observed between the development and validation cohorts (ΔAUC = −0.0042 for undernutrition and +0.0016 for overnutrition) is a critical finding for model credibility. In contrast, GROW and INTERGROWTH-21st showed substantially greater AUC degradation (ΔAUC = −0.027 to −0.044), suggesting that the cFBMI model generalizes better across time periods. This temporal stability is consistent with the model’s coefficient of variation (CV = 9.7%) being substantially lower than that of GROW (CV = 12%), reflecting greater precision in the underlying growth reference. Stable Brier scores across cohorts further support the absence of meaningful overfitting.

### 4.5. Calibration and Clinical Implications

The calibration analysis revealed that cFBMI produced observed proportions (9.6%/81.7%/8.8%) closely approximating the expected 10%/80%/10% distribution, with the smallest chi-square deviation of the three methods (χ^2^ = 9.22, *p* = 0.010). Good calibration is essential for clinical use: a well-calibrated model ensures that when a neonate is classified as undernourished (PI < p10), the predicted probability reflects the true likelihood of nutritional compromise. The superior calibration of cFBMI, combined with its higher discrimination, supports further prospective clinical evaluation of the model as a potential tool for neonatal nutritional status assessment.

### 4.6. Decision Curve Analysis

The decision curve analysis confirmed the clinical utility of the cFBMI model across a wide range of threshold probabilities for both undernutrition and overnutrition detection. At all clinically relevant thresholds, cFBMI provided greater net benefit than GROW, INTERGROWTH-21st, and the default strategies of treating all or none. This finding is particularly important because DCA directly quantifies the clinical consequences of using a model, accounting for the trade-off between true positives and false positives [16,23], a methodology our group has previously applied in the external validation of clinical prediction models in different settings [30]. The greater net benefit of cFBMI across threshold probabilities suggests that further prospective clinical evaluation is warranted to assess whether its application could lead to more appropriate identification of neonates requiring nutritional intervention while minimizing unnecessary interventions. The present results should therefore be interpreted as a proof-of-concept framework for future clinical validation rather than as evidence supporting immediate clinical implementation.

### 4.7. Prenatal vs. Postnatal Application—Conceptual and Clinical Implications

The conceptual contribution of the cFBMI framework is the introduction of fetal length as a constitutive dimension of the fetal nutritional-assessment construct. Current obstetric practice operationalizes fetal nutritional status almost exclusively through fetal weight, via ultrasound-estimated fetal weight (EFW) compared against weight-only customized (GROW) or population (INTERGROWTH-21st [17] references. Fetal length is not routinely estimated, measured, or integrated into clinical decision making about fetal nutritional status. The cFBMI framework proposes that nutritional status is properly defined by proportionality (weight relative to length), and provides a customized, validated operational vehicle for evaluating this construct across the perinatal continuum.

An important consideration for clinical implementation is the distinction between prenatal and postnatal application of the cFBMI model. In the present validation study, the observed BMI at birth was calculated using actual neonatal birthweight and birth length, representing the postnatal scenario, in which the measured length contributes independent discriminative information that the cFBMI exploits to achieve an AUC > 0.96 against the Ponderal Index reference standard. In prenatal application—where fetal length is estimated from maternal characteristics and gestational age via the cFBMI’s regression equation—the L_est term cancels algebraically between BMI_obs and BMI_est, and the prenatal cFBMI z-score reduces to (EFW/W_est − 1)/0.097. The prenatal cFBMI ranking is therefore rank-correlated with—but not identical to—the GROW customized birthweight ranking, because the cFBMI’s expected weight regression differs from Gardosi’s customized birthweight regression used by GROW, and because the coefficient of variation of cFBMI (CV = 9.7%) differs from that of GROW (CV = 12%). For any given fetus, the two methods may therefore assign different W_est values and, after CV scaling, different operational classifications relative to the standard p10/p90 thresholds.

A biological consideration further supports the prenatal architecture of the cFBMI. In placental insufficiency—the dominant etiology of pathological intrauterine growth restriction [20,31]—fetal weight is preferentially affected while fetal length is comparatively preserved (the classical asymmetric-IUGR phenotype). In this clinically dominant scenario, the true prenatal fetal length closely approximates the theoretical L_est predicted by the cFBMI regression, so that the substitution of L_est for L_real in the prenatal BMI_obs introduces only modest approximation error. The cancellation of L_est therefore reflects, rather than discards, the biology of fetal nutritional deprivation: the discriminative information is appropriately concentrated in the ratio EFW/W_est, which captures the nutritionally responsive dimension. In symmetric IUGR—the rarer phenotype, in which length is also restricted—the cancellation remains clinically appropriate, because the preserved proportionality yields a normal BMI z-score (constitutional smallness rather than malnutrition).

The translational contribution of the cFBMI model should therefore be understood as follows. First, the framework “degrades gracefully” to weight-anchored assessment when length information is unavailable, and outperforms weight-based methods substantially (ΔAUC ≈ 0.21–0.28) when measured length becomes available at birth. Second, the primary clinical decision-making locus for neonatal nutritional assessment is the moment of delivery and the perinatal period, at which length is directly measurable. Third, the framework is forward-compatible with emerging modalities for independent fetal length estimation (3D ultrasound surface reconstruction, MRI fetometry, AI-enhanced femur-to-crown-heel conversion); once any of these is validated and integrated, the partial algebraic cancellation of L_est breaks and the discriminative gain extends into the antenatal phase. Fourth, BMI is the universal nutritional metric across the lifespan, and the cFBMI framework therefore enables longitudinal continuity from fetal to neonatal to pediatric care that weight-based customized methods, by construction, cannot provide.

In summary, the cFBMI is not proposed as an algorithmic replacement for GROW in the strict prenatal-only setting, where the rankings of the two methods are highly correlated. It is proposed as a conceptual reframing of fetal nutritional assessment—from weight-only to proportionality—operationalized in a customized model whose primary discriminative advantage is empirically realized at and beyond the moment of birth, and which provides a coherent vehicle for the integration of independent fetal length information as antenatal imaging matures.

### 4.8. Comparison with Prior Validation and Literature

The present results are consistent with those of the initial validation of the cFBMI model in the general obstetric population [11], where AUCs of 0.962 (SGA) and 0.941 (LGA) were reported. The temporal stability observed here (ΔAUC ≤ 0.004) confirms that the model’s performance is not an artifact of the development data but reflects a genuine biological signal captured by the integration of weight and length. Compared with published studies using GROW or INTERGROWTH-21st for nutritional classification, the cFBMI model demonstrates substantially superior discrimination, consistent with the theoretical advantage of incorporating body proportionality [6,7].

### 4.9. Strengths

The principal strengths of this study include: (1) the temporal validation design with strictly non-overlapping cohorts (2002–2021 development; 2022–2025 validation), eliminating data leakage; (2) adherence to the Riley et al. framework for external validation reporting; (3) comprehensive evaluation, including discrimination, calibration, clinical utility via DCA, and temporal stability metrics; (4) a large validation cohort (*n* = 4441 singleton deliveries); (5) comparison against two well-established reference methods (GROW customized birthweight and INTERGROWTH-21st); and (6) use of actual birth measurements (weight and length) rather than ultrasound estimates, eliminating measurement error from fetal biometry.

### 4.10. Limitations and Future Directions

This study has several important limitations that must be considered when interpreting the findings and planning future research.

#### 4.10.1. Temporal Validation Within a Single Institution

Both the development cohort (2002–2021, *n* = 7864) and validation cohort (2022–2025, *n* = 4441) were derived from the same tertiary referral center. This represents temporal rather than geographic external validation. The shared institutional context (protocols, equipment, clinical practices, patient population) means that performance in truly external populations—with different ethnic compositions, healthcare systems, measurement protocols, or socioeconomic contexts—remains unestablished. External multicenter, prospective validation across geographically diverse populations is an essential prerequisite before clinical implementation [12]. Furthermore, it does not test the model’s transportability across different institutions, populations, or clinical protocols. Geographic and domain validation studies are therefore required as the next step in the validation hierarchy.

#### 4.10.2. Selection Bias from Data Completeness Requirements

The 19.1% exclusion rate (1047 of 5488 deliveries excluded in the validation cohort) introduces potential selection bias. Excluded cases had higher rates of preterm birth, maternal obesity, and pregnancy complications, suggesting the included population may not represent the full clinical spectrum. Performance in high-risk populations and cases with incomplete data may differ from reported estimates. Future studies should develop strategies for handling missing data (e.g., multiple imputation) to maximize real-world applicability. The exclusion rate of 19.1% (1047/5488 deliveries) is primarily attributable to missing maternal anthropometric data (*n* = 744, 71% of exclusions). A comparison of the included and excluded populations would help quantify potential selection bias; however, the nature of the exclusion (administrative data incompleteness) makes it likely that missing data are related to logistic factors (e.g., emergency admissions, incomplete antenatal records) rather than to neonatal nutritional status itself. Nevertheless, the generalizability of the results to populations with high rates of incomplete data should be interpreted with caution. Future implementation studies should incorporate multiple imputation strategies to maximize clinical applicability.

#### 4.10.3. Verification Bias

Stillbirths and neonatal deaths were excluded from the analysis due to absence of postnatal anthropometric measurements. These cases likely represent the most severe end of the nutritional status spectrum, and their exclusion may artificially inflate the discriminative performance by removing extreme cases. Future studies should develop methods to include these outcomes, potentially using estimated rather than measured postnatal anthropometry.

#### 4.10.4. Gold Standard Limitations

The Ponderal Index (PI = weight/length^3^) and Metcoff score, while widely used, are imperfect proxies for nutritional status. The PI has limited sensitivity for detecting undernutrition in preterm neonates and does not distinguish between symmetric and asymmetric growth restriction. The Metcoff score reliability depends on the experience of the examining clinician. Validation against more objective measures (DEXA-measured body composition, bioelectrical impedance) would strengthen the evidence base. This mathematical coupling between the index test and the reference standard is a fundamental limitation of the present study. While it reflects the deliberate design choice of evaluating a proportionality-based model against a proportionality-based gold standard, readers should interpret the absolute AUC values with this caveat in mind. Validation against independent clinical endpoints—rather than anthropometric surrogates—is required to establish the clinical relevance of these findings. Importantly, however, all three classifiers under comparison share input variables with the Ponderal Index reference standard, yet only cFBMI achieves AUC values in the 0.95+ range; the differential performance therefore reflects the discriminative contribution of measured birth length rather than mathematical coupling per se. In an internal pilot study from our group (*n* = 116; reference standard: Clinical Assessment of Nutritional Status [CAN] score < 25; manuscript in preparation), the cFBMI retained a statistically significant sensitivity advantage over both INTERGROWTH-21st and an institutional customized birthweight model (67.7% vs. 45.2% and 51.6%, *p* < 0.001) at the standard p10 threshold, against a reference standard sharing no input variable with any index test.

#### 4.10.5. Measurement Error in Ultrasound Estimates

The regression models used to estimate expected weight and length carry inherent prediction error. These errors propagate into cFBMI calculations and may introduce non-differential misclassification. However, in the postnatal setting (as in this study), actual birth weight and birth length are used for BMI_obs, eliminating measurement error from fetal biometry estimation. Prospective validation using prenatal model-estimated cFBMI (rather than birth measurements) is needed to assess performance in the antenatal setting.

#### 4.10.6. Absence of Clinical Outcome Data

This study validated cFBMI against anthropometric proxies (PI, Metcoff score) rather than clinical outcomes (neonatal morbidity, NICU admission, neurodevelopmental outcomes). While anthropometric validation is a necessary first step, clinical outcome validation is ultimately required to demonstrate that improved nutritional status detection translates into improved patient outcomes. Future studies should link cFBMI classification to neonatal morbidity endpoints.

#### 4.10.7. Implementation Feasibility

The postnatal cFBMI calculation requires actual birth length measurement, which is subject to measurement error in clinical practice. Prenatal implementation would use model-estimated weight and length from maternal characteristics and, as discussed in Section 2.3.4, the algebraic cancellation between estimated length in BMI_obs and BMI_est would reduce the prenatal cFBMI to a weight-only assessment equivalent to GROW. Prospective studies evaluating the feasibility and performance of prenatal cFBMI calculation are needed before clinical implementation protocols can be developed.

## 5. Conclusions

This temporal validation study provides promising preliminary evidence that the customized fetal body mass index percentile model demonstrates excellent discriminative performance (c-statistic > 0.96 for both undernutrition and overnutrition detection), superior calibration, and apparent advantages in clinical utility as determined by decision curve analysis compared with the GROW customized birthweight model and the INTERGROWTH-21st population standard, in an independent temporal validation cohort of 4441 singleton deliveries (2022–2025). The minimal AUC degradation between the development and validation cohorts (ΔAUC ≤ 0.004) confirms temporal stability and a low overfitting risk. The model’s integration of body proportionality provides fundamental conceptual alignment with the Ponderal Index gold standard, explaining its superior performance over weight-only methods.

However, these findings must be interpreted with appropriate caution. This study represents temporal validation within a single institution; both development and validation cohorts share the same institutional protocols, ultrasound equipment, and clinical practices. Performance in truly external populations—with different ethnic compositions, healthcare systems, measurement protocols, or clinical contexts—remains to be established. Additionally, the exclusion of cases with incomplete data introduces potential selection bias, meaning the results may not reflect real-world performance.

External multicenter, prospective validation across geographically diverse populations is an essential prerequisite before broad clinical implementation of the cFBMI model can be recommended. Future studies should also validate the model against direct body composition measures and clinical outcomes (neonatal morbidity, NICU admission) and develop strategies for handling missing data to maximize real-world applicability. Subject to these validations, the cFBMI model represents a promising investigational tool for neonatal nutritional status assessment that merits further rigorous evaluation.

## Figures and Tables

**Table 1:** Demographic and clinical characteristics of the development and temporal validation cohorts. Original cohort values reproduced from Fernández Alba et al. (Diagnostics 2025, 15, 877) [11]. Continuous variables: median (IQR) or mean (SD) as originally reported. Categorical variables: n (%).

Variable	Development Cohort (*n* = 9499) January 2011–December 2021	Validation Cohort (*n* = 4441) January 2022–December 2025
Neonatal birthweight (g)	3282.65 (435.19)	3280.0 (2980.0–3595.0)
Neonatal birth length (cm)	50 (3)	50.0 (48.0–51.0)
Gestational age (days)	275 (9.8)	277.0 (271.0–283.0)
Neonatal sex		
Male	4859 (51%)	2261 (50.9%)
Female	4640 (49%)	2180 (49.1%)
Maternal pre-pregnancy weight (kg)	62 (12)	65.0 (58.0–76.0)
Maternal height (cm)	162 (9)	163.0 (159.0–167.0)
Maternal age (years)	32.22 (7.16)	33.0 (29.1–36.6)
Mode of delivery		
Cesarean section	1914 (20%)	1167 (26.3%)
Vaginal delivery	7585 (80%)	3274 (73.7%)
Parity		
Nulliparous (para 0)	5744 (61%)	2716 (61.2%)
Para 1	2973 (31%)	1330 (29.9%)
Para ≥ 2	782 (8%)	395 (8.9%)
Gravidity		
Gravida 1	3989 (42%)	1744 (39.3%)
Gravida 2	4275 (35%)	1480 (33.3%)
Gravida ≥ 3	2185 (23%)	1217 (27.4%)
Previous miscarriage		
None	7517 (79%)	3296 (74.2%)
1	1412 (15%)	840 (18.9%)
≥2	570 (6%)	304 (6.8%)

Continuous variables reported as mean (SD) for original cohort (as published) and median (IQR) for validation cohort. Categorical variables: n (%).

**Table 2:** Discrimination metrics for undernutrition detection (Ponderal Index < p10) in the temporal validation cohort (*n* = 4441). AUC: area under the ROC curve; PPV: positive predictive value; NPV: negative predictive value; LR+: positive likelihood ratio; LR−: negative likelihood ratio; DOR: diagnostic odds ratio; κ: Cohen’s kappa. Bootstrap 95% CI (2000 iterations).

Metric	cFBMI	GROW	INTERGROWTH-21st
AUC (95% CI)	0.9619 (0.952–0.972)	0.7509 (0.728–0.773)	0.7557 (0.733–0.778)
Sensitivity	0.55	0.27	0.25
Specificity	0.99	0.95	0.95
PPV	0.87	0.49	0.47
NPV	0.92	0.88	0.88
LR+	37.73	5.40	4.97
LR−	0.46	0.77	0.79
DOR	82.87	7.01	6.31
Cohen’s κ	0.61	0.18	0.17
DeLong vs. cFBMI (*p*)	—	<0.0001	<0.0001

**Table 3:** Discrimination metrics for overnutrition detection (Ponderal Index > p90) in the temporal validation cohort (*n* = 4441). Abbreviations as in Table 2.

Metric	cFBMI	GROW	INTERGROWTH-21st
AUC (95% CI)	0.9612 (0.950–0.972)	0.6761 (0.648–0.704)	0.6822 (0.655–0.709)
Sensitivity	0.62	0.28	0.27
Specificity	0.97	0.90	0.90
PPV	0.72	0.24	0.23
NPV	0.96	0.92	0.92
LR+	22.37	2.75	2.71
LR−	0.39	0.80	0.81
DOR	56.92	3.43	3.34
Cohen’s κ	0.61	0.18	0.17
DeLong vs. cFBMI (*p*)	—	<0.0001	<0.0001

AUC: area under the ROC curve; PPV: positive predictive value; NPV: negative predictive value; LR+: positive likelihood ratio; LR−: negative likelihood ratio; DOR: diagnostic odds ratio; κ: Cohen’s kappa (three-class agreement: SGA/AGA/LGA vs. Ponderal Index gold standard). Bootstrap 95% CI (2000 iterations).

**Table 4:** Temporal stability: AUC and Brier score comparison between development (2002–2021) and temporal validation (2022–2025) cohorts for undernutrition (PI < p10) and overnutrition (PI > p90) detection. ΔAUC = AUC(val) − AUC(dev).

Method	AUC Dev	AUC Val	ΔAUC	Brier Dev	Brier Val	ΔBrier
Undernutrition (PI < p10)						
cFBMI	0.9661	0.9619	−0.0042	0.2284	0.2275	−0.0009
GROW	0.7779	0.7509	−0.0270	0.2519	0.2539	+0.0020
INTERGROWTH-21st	0.7863	0.7557	−0.0306	0.2456	0.2441	−0.0015
Overnutrition (PI > p90)						
cFBMI	0.9596	0.9612	+0.0016	0.2601	0.2305	−0.0296
GROW	0.7201	0.6761	−0.0440	0.3260	0.3183	−0.0077
INTERGROWTH-21st	0.7255	0.6822	−0.0433	0.3122	0.3115	−0.0007

## Data Availability

The data supporting the reported results are available from the corresponding author upon reasonable request, subject to applicable data protection regulations.
